# Correction: Shrapnel, W.S.; Butcher, B.E. Sales of Sugar-Sweetened Beverages in Australia: A Trend Analysis from 1997 to 2018. *Nutrients* 2020, *12*, 1016

**DOI:** 10.3390/nu13041356

**Published:** 2021-04-19

**Authors:** William S. Shrapnel, Belinda E. Butcher

**Affiliations:** 1 Shrapnel Nutrition Consulting Pty Ltd., 790 Pinnacle Road, Orange, NSW 2800, Australia; 2 WriteSource Medical Pty Ltd., Lane Cove, NSW 2066, Australia; bbutcher@writesourcemedical.com.au; 3 School of Medical Science, University of New South Wales (UNSW), Sydney, NSW 2052, Australia

The authors wish to make the following corrections to this paper [1]. Following publication, it was discovered that the data provided to the authors for fruit juice was incorrect. Updated data were provided by IRI Australia. This affects the text relating to Figure 3 and the juice totals for Table 1. Corrected data are listed below.

**3. Results:** Data on per capita volume sales for juice, juice drinks and flavoured milks were only available for the period 2009 to 2018. During this period, volume sales of juice drinks (<100% juice) declined slightly, while sales of 100% juice dropped sharply, by 43% (Figure 3).

**Table 1:** Trends in volume sales of water-based beverages between 1997 and 2018. * Total may not equal sum of sugar-sweetened beverages (SSB) and non-SSB categories due to rounding error.

Beverage Category	Per Capita Volume Sales 1997 (L/Person)	Per Capita Volume Sales 2009 (L/Person)	Per Capita Volume Sales 2018 (L/Person)	Volume Share 1997 (%) *	Volume Share 2018 (%) *	Percentage Change per Capita Volume Sales 2009–2018 (%)	Annual Growth Rate 2009–2018 (%)	Percentage Change per Capita Volume Sales 1997–2018 (%)	Annual Growth Rate 1997–2018 (%)
All water-based beverages *									
Total	130.86	129.61	149.42	100.00	100.00	0.67	1.53	14.18	0.64
SSB	83.16	74.40	61.04	63.54	40.85	−0.82	−1.80	−26.59	−1.21
Non-SSB	47.71	55.21	88.38	36.45	59.15	3.27	6.01	85.26	3.88
Carbonated soft drinks									
Total	98.54	91.62	70.58	75.30	47.24	−0.79	−2.30	−28.37	−1.29
SSB	75.78	62.51	44.62	57.91	29.86	−1.69	−2.86	−41.13	−1.87
Non-SSB	22.76	29.12	25.97	17.39	17.37	2.21	−1.08	14.08	0.64
Energy drinks									
Total	0.14	3.56	6.36	0.11	1.33	81.39	7.86	4374.72	198.85
SSB	0.14	3.30	5.44	0.11	1.16	73.61	6.50	3728.39	169.47
Non-SSB	0.00	0.26	0.92	0.00	0.17	NC	25.07	NC	NC
Sports drinks									
Total	1.48	2.79	3.97	1.13	2.66	7.29	4.24	168.25	7.65
SSB	1.48	2.70	3.78	1.13	2.53	7.18	4.03	156.05	7.09
Non-SSB	0.00	0.09	0.19	0.00	0.13	61.48	10.57	6478.99	294.50
Iced tea									
Total	0.15	1.30	1.99	0.16	1.26	44.84	5.30	1217.43	55.34
SSB	0.15	1.15	1.73	0.16	0.78	39.36	5.09	1043.63	47.44
Non-SSB	0.00	0.16	0.26	0	0.56	NC	6.88	NC	NC
Mineral waters									
Total	5.00	4.49 ^†^	9.02 ^†^	3.82	6.03 ^†^	−0.58	10.08	80.47	3.66
SSB	2.17	1.49	1.88	1.66	1.26	−2.27	2.61	−13.47	−0.61
Non-SSB	2.82	0.80	1.17	2.61	2.01	−6.02	4.51	−58.65	−2.67
Mixers									
Total	7.97	7.24	8.36	6.09	5.59	−0.07	1.55	4.89	0.22
SSB	3.41	2.48	3.01	2.61	2.01	9.14	2.11	−11.88	−0.54
Non-SSB	4.55	4.75	5.35	3.48	3.58	−6.98	1.25	17.47	0.79
Unflavoured pure waters									
Total	11.81	20.02	53.88	9.02	36.06	4.32	16.91	356.33	16.20
Still	5.76	17.83	47.91	4.41	32.57	14.32	16.88	732.37	33.29
Sparkling	-	2.19	5.97	-	3.99	NC	17.21	NC	NC
Flavoured Milks									
Total	-	6.06	9.48	-	-	56.42	5.64	NC	NC
Juice									
Total	-	362.59	230.75	-	-	−36.36	−3.64	NC	NC
100% Juice	-	257.21	133.83	-	-	−47.97	−4.80	NC	NC
Less than 100% Juice	-	105.38	96.93	-	-	−8.03	−0.80	NC	NC
Kombucha									
Total	-	0.00	0.47	-	-	NC	NC	NC	NC

The correct data were provided by IRI Australia. Please replace the above table with this one below.

**Table 1:** Trends in volume sales of water-based beverages between 1997 and 2018. * Total may not equal sum of SSB and non-SSB categories due to rounding error.

Beverage Category	Per Capita Volume Sales 1997 (L/Person)	Per Capita Volume Sales 2009 (L/Person)	Per Capita Volume Sales 2018 (L/Person)	Volume Share 1997 (%) *	Volume Share 2018 (%) *	Percentage Change per Capita Volume Sales 2009–2018 (%)	Annual Growth Rate 2009–2018 (%)	Percentage Change per Capita Volume Sales 1997–2018 (%)	Annual Growth Rate 1997–2018 (%)
All water-based beverages *									
Total	130.86	129.61	149.42	100.00	100.00	0.67	1.53	14.18	0.64
SSB	83.16	74.40	61.04	63.54	40.85	−0.82	−1.80	−26.59	−1.21
Non-SSB	47.71	55.21	88.38	36.45	59.15	3.27	6.01	85.26	3.88
Carbonated soft drinks									
Total	98.54	91.62	70.58	75.30	47.24	−0.79	−2.30	−28.37	−1.29
SSB	75.78	62.51	44.62	57.91	29.86	−1.69	−2.86	−41.13	−1.87
Non-SSB	22.76	29.12	25.97	17.39	17.37	2.21	−1.08	14.08	0.64
Energy drinks									
Total	0.14	3.56	6.36	0.11	1.33	81.39	7.86	4374.72	198.85
SSB	0.14	3.30	5.44	0.11	1.16	73.61	6.50	3728.39	169.47
Non-SSB	0.00	0.26	0.92	0.00	0.17	NC	25.07	NC	NC
Sports drinks									
Total	1.48	2.79	3.97	1.13	2.66	7.29	4.24	168.25	7.65
SSB	1.48	2.70	3.78	1.13	2.53	7.18	4.03	156.05	7.09
Non-SSB	0.00	0.09	0.19	0.00	0.13	61.48	10.57	6478.99	294.50
Iced tea									
Total	0.15	1.30	1.99	0.16	1.26	44.84	5.30	1217.43	55.34
SSB	0.15	1.15	1.73	0.16	0.78	39.36	5.09	1043.63	47.44
Non-SSB	0.00	0.16	0.26	0	0.56	NC	6.88	NC	NC
Mineral waters									
Total	5.00	4.49^†^	9.02^†^	3.82	6.03^†^	−0.58	10.08	80.47	3.66
SSB	2.17	1.49	1.88	1.66	1.26	−2.27	2.61	−13.47	−0.61
Non-SSB	2.82	0.80	1.17	2.61	2.01	−6.02	4.51	−58.65	−2.67
Mixers									
Total	7.97	7.24	8.36	6.09	5.59	−0.07	1.55	4.89	0.22
SSB	3.41	2.48	3.01	2.61	2.01	9.14	2.11	−11.88	−0.54
Non-SSB	4.55	4.75	5.35	3.48	3.58	−6.98	1.25	17.47	0.79
Unflavoured pure waters									
Total	11.81	20.02	53.88	9.02	36.06	4.32	16.91	356.33	16.20
Still	5.76	17.83	47.91	4.41	32.57	14.32	16.88	732.37	33.29
Sparkling	-	2.19	5.97	-	3.99	NC	17.21	NC	NC
Flavoured Milks									
Total	-	6.06	9.48	-	-	56.42	5.64	NC	NC
Juice									
Total	-	36.70	24.62	-	-	−32.92	−3.29	NC	NC
100% Juice	-	26.0	18.82	-	-	−43.02	−4.30	NC	NC
Less than 100% Juice	-	10.69	9.80	-	-	−8.34	−0.83	NC	NC
Kombucha									
Total	-	0.00	0.47	-	-	NC	NC	NC	NC

**Figure 3:**
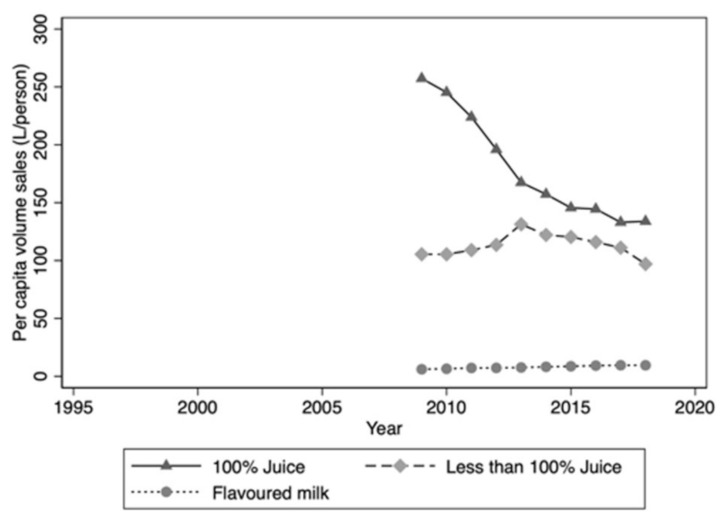
Per capita volume sales: Juice/juice drinks and flavoured milk drinks 2009–2018.

The correct data were provided by IRI Australia. Please replace the above figure with this one below.

**Figure 3:**
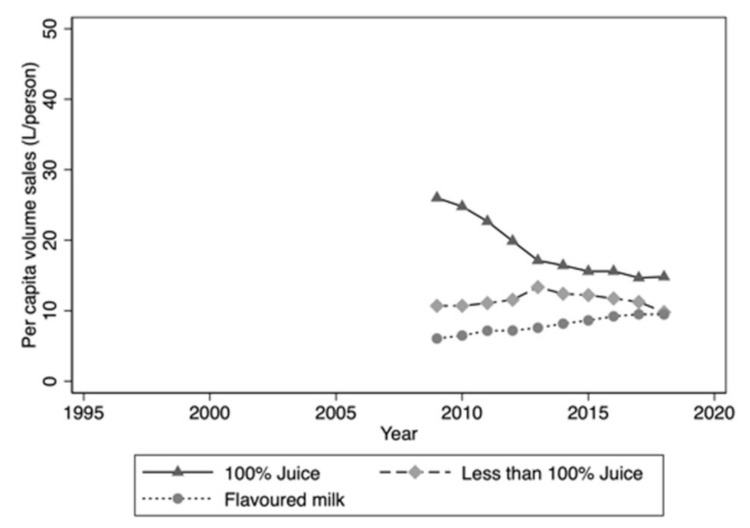
Per capita volume sales: Juice/juice drinks and flavoured milk drinks 2009–2018.

These changes have no material impact on the conclusions of the paper. The authors would like to apologize to readers of *Nutrients* for this error. The published version will be updated on the article webpage, with a reference to this correction notice.

## Data Availability

Data available on a commercial basis from IRI Australia.
